# Supplementary material for: Genetic Characteristics of the Rat Fibroblast Cell Line Rat-1
Source: Cells. 2024 Dec 29;14(1):21. doi: 10.3390/cells14010021 (PMC11719652; doi:10.3390/cells14010021)
Supplement: Supplementary file 1 [file cells-14-00021-s001.zip › Figure S1.pdf]

Supplemental Figures

Genetic Characteristics of the Rat Fibroblast Cell Line Rat-1

Thomas Liehr [1,*], Stefanie Kankel [1], Eva Miriam Buhl [2], Sarah K. Schröder-Lange [3], and Ralf Weiskirchen [3,*]

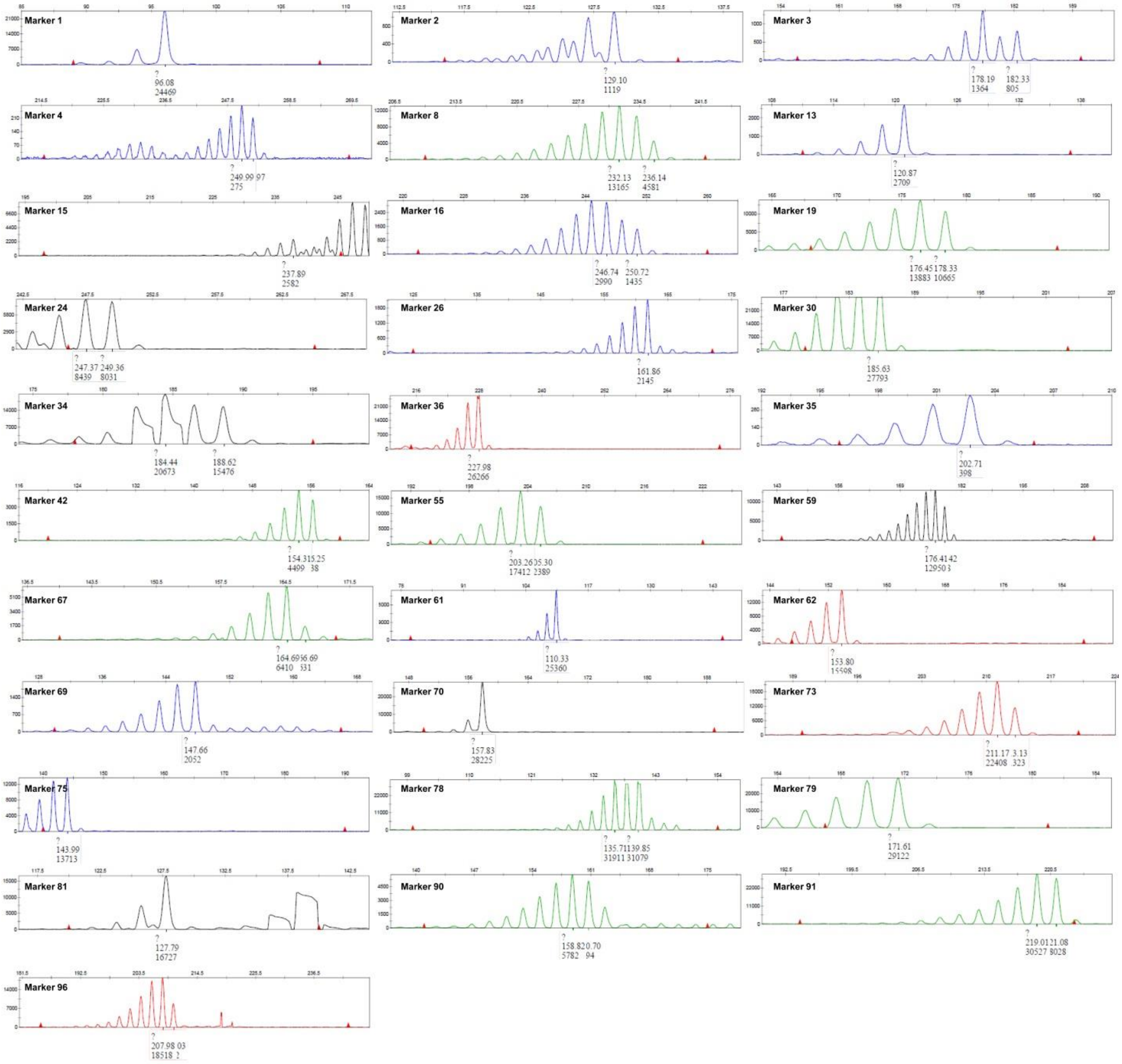

Figure S1. Short Tandem Repeat (STR) profile chromatograms for 31 variant markers of Rat-1 cells. These chromatograms display the amplification results of each dinucleotide repeat marker in the CellCheck™ Rat system. The peaks on the x-axis indicate different allele sizes, with the height of the peak corresponding to the number of alleles. Each marker is assigned a number, and the allele sizes are measured in base pairs (bp).
